# Single molecule iSCAT imaging reveals a fast, energy efficient search mode for the DNA repair protein UvrA†

**DOI:** 10.1039/d1nr06913f

**Published:** 2022-03-21

**Authors:** Robert J. Charman, Neil M. Kad

**Affiliations:** School of Biological Sciences, Division of Natural Sciences, University of Kent Canterbury CT2 7NH UK n.kad@kent.ac.uk +44 (0)1227 816151

## Abstract

Exposure to UV radiation results in numerous DNA lesions, which threaten genome integrity. The nucleotide excision DNA repair pathway detects and repairs a range of such UV-induced DNA lesions. In bacteria, initial damage detection and verification is carried out by two proteins: UvrA and UvrB. Despite decades of study, the process of how these proteins locate damage remains unclear. Here we use high-speed interferometric scattering (iSCAT) microscopy, in combination with a surface-bound-DNA assay, to investigate early damage detection by UvrA. We have discovered that UvrA interacts with DNA in two phases; a slow phase (∼1.3 s^−1^) that correlates with an ATP-consuming state previously identified, and a second, much faster search mode. These faster interactions persist for ∼130 ms and using ATP analogues we determine this phase does not require ATP consumption. Including this new fast-search state in a model of the DNA search process reveals that only with this state is it possible for basal levels of UvrA to explore 99% of the *E. coli* genome within a single division cycle. Altogether, this work uncovers the presence of a rapid, energy efficient search mechanism, which allows UvrA alone to search the entirety of the *E. coli* genome within a single division cycle.

## Abstract 2

Rapid interactions of single UvrA-Qdot complexes can be detected binding to DNA affixed to a surface using iSCAT imaging.

## Graphical abstract

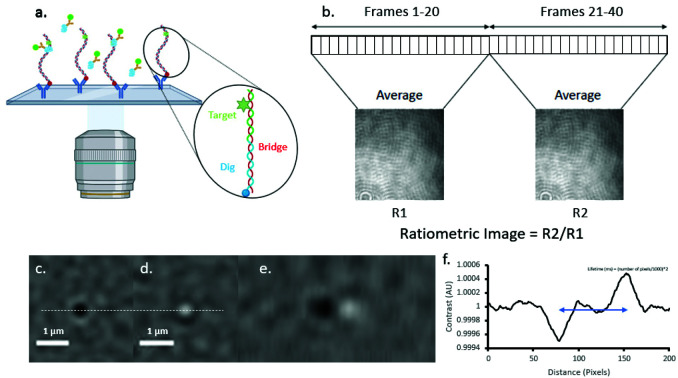

## Introduction

The genome is constantly being damaged by both endogenous and exogenous sources; to combat this a wide range of DNA repair mechanisms have evolved to target and repair damage. Nucleotide excision repair (NER) is a highly conserved DNA repair mechanism that targets a wide variety of bulky DNA lesions,^1^ such as 6-4 photoproducts and cyclobutane pyrimidine dimers resulting from UV irradiation, in addition to artificial damage such as fluorescein.^2–4^ The process of NER can be dissected into discrete steps: damage detection, damage verification, incision, excision, DNA synthesis, and ligation.^5^ Damage detection involves the collaborative work of the UvrA and UvrB proteins, binding DNA either as a UvrAB complex^6,7^ or sequentially.^6,8^ The UvrB helicase separates the two strands and inserts a beta-hairpin for damage verification, leading to the release of UvrA. UvrC is then recruited to the damage site and using its two nuclease domains is able to make incisions either side of the DNA lesion.^7^ UvrBC (the post-incision complex) is then displaced by UvrD and DNA polymerase I, excising the nicked fragment, and subsequently resynthesizing the strand using the opposite strand as a template. DNA ligase is then recruited to ligate the fragment, completing the pathway.^9^

UvrA is the first responder to damage in the NER pathway^9^ and forms a dimer.^10^ Each monomer possesses two complete ATP-binding domains, each containing an A-Walker motif site, a Q-loop for nucleotide binding, a Walker B motif, a D-loop, an ABC signature domain, and a his-loop. Additional domains are also located within the N-terminal ATP-binding domain, with one being involved in binding UvrB,^10,11^ and also the insertion domain for binding DNA.^12^ The UvrA ATPase mechanism is complex with numerous intermediates,^13^ including allosteric communication between sites, which couples the binding of UvrA to lesion with loading of UvrB, most likely as a consequence of turnover at the proximal ATPase site.^8,13–15^

The search for damaging lesions by DNA repair systems presents a considerable challenge, due to the vast quantity of non-target DNA which has to be searched by a relatively small number of proteins. Numerous mechanisms of search have been suggested^16,17^ that permit rapid and in some cases faster than 3D diffusional search. Included in this are scenarios beginning with non-specific attachment of proteins to DNA, followed by 1D sliding along the DNA, hopping along the DNA backbone, intersegmental DNA transfers, and hopping to different DNA strands.^18–21^ UvrA has been previously shown to use a 3-dimensional search mechanism,^6^ in which molecules undergo Brownian diffusion within the cell, and upon interaction with DNA UvrA remains static for ∼1–2 s.^8,14^ However, as previously noted this 3D-only search mechanism alone would be incapable of searching an *Escherichia coli* genome (4.6 × 10^6^) base pairs (bp) within a single division cycle, only being able to effectively search 12% of the genome in this time.^6^ Furthermore, since each interaction of UvrA_2_ with the DNA consumes 2 molecules of ATP,^13,14^ this represents a considerable usage of the cell's energy resources. In association with UvrB, UvrA is able to form the UvrA_2_B_2_ complex which collapses the 3D search of UvrA into a 1D search.^6^ This dramatically increases the speed of search, subsequently increasing the likelihood of locating DNA lesions. However, *in vivo* work has found that ∼90% of UvrB within the cell remains diffusive within the cytoplasm and is instead recruited by damage-bound UvrA from solution.^8^ Therefore, to search effectively UvrA must locate damage first, suggesting that an alternative, faster, ATP-independent mechanism of search may exist.

In this study, we use iSCAT microscopy which provides a useful alternative to fluorescence imaging for the investigation of UvrA–DNA interactions. iSCAT is based on the detection of light scattered by nanoscopic particles. This is achieved through the imaging of the interference that occurs between this scattered light and a reference wave, consisting of light reflected at the glass-water interface of the coverslip.^22,23^ Due to the non-degradative nature of light scattering, theoretically unlimited imaging times can be achieved, with the achievable temporal resolution only being limited by the power of the illuminating laser. This advantage, in combination with the extreme sensitivity of iSCAT, has allowed for high-speed unlabelled imaging of proteins,^27^ and microsecond tracking of diffusion within cell membranes,^24,25^ and purely scattering based mass photometry of proteins with a resolution of 19 kDa.^26^

Here we investigate the existence of a more rapidly interacting species using interferometry-based detection of UvrA–DNA interactions. This methodology provides exceptionally high temporal and spatial resolution, theoretically unlimited imaging time, and straightforward identification of protein complex stoichiometry.^27,28^ In combination with a surface-based assay allowing for facile introduction of synthetic adducts onto surface-immobilised double-stranded DNA, we are able to study UvrA interactions with both damaged and undamaged DNA on a millisecond time scale. In this time regime we can detect binding events that occur faster than ATP turnover, revealing a novel binding mechanism through which UvrA may be able to detect damage more efficiently using a non-ATP consuming rapid search.

## Methods

### Interferometric scattering microscopy

The system described in ESI Fig. 1† used the output of a 300 mW 445 nm diode laser (Roithner-Lasertechnik ULV-445-300), modulated at 20 kHz, focused through a 50 μm pinhole filter (ThorLabs P50HD) *via* a 100 mm plano-convex NBK-7 lens. This beam was recollimated *via* a 200 mm plano-convex NBK-7 lens, before being focused into the back focal plane of the objective (Nikon CFI Apochromat TIRF 60× oil immersion objective lens, NA 1.46) by a 200 mm plano-convex NBK-7 lens. The beam was redirected to the objective using a 50 : 50 beam splitter, providing separation between the illumination and detection pathways. The resulting reflected and scattered fields are collected by the objective, before travelling back through the 50 : 50 beam splitter, and collimated by a 200 mm plano-convex NBK-7 lens. The reference and scattered waves pass through a telescope constructed from a 50 mm plano-convex NBK-7 lens and a 150 mm plano-convex NBK-7 lens, resulting in a final magnification of 180x on the sensor of a Flir Grasshopper USB3 CMOS camera (Point Grey Research, GS3-U3-23S6M-C). The resulting field of view was 20 μm × 20 μm, with a pixel size of 37.5 nm. Fine XYZ translation of the sample is achieved using a 3-axis Nano-LPS200 (MadCityLabs), controlled by a 3-drive Nano-Drive (MadCityLabs), this stage is then mounted on a PT3-XYZ translation block (ThorLabs) to allow for manual coarse adjustment of XYZ positions. The system was housed within an isolated air-conditioned room (held at 20 °C) and enclosed with internal compartmentalisation to mitigate any potential thermal drift. The maximum positional drift in X and Y was <37.5 nm. All images were acquired at 500 fps, for a total of 30 000 frames per video.

### Oligonucleotide list and construct design

The damaged_98 bp and undamaged_98 bp constructs are formed from 3 oligonucleotides: digoxigenin-labelled oligonucleotide, bridge oligonucleotide, and either F26,50 oligonucleotide or undamaged F26,50 oligonucleotide (for damaged and undamaged respectively). The bridge oligonucleotide is complementary to both the digoxigenin-labelled oligonucleotide and the (undamaged) F26,50 oligonucleotide, providing a 96 bp region of dsDNA. The fluorescein modification on the F26,50 oligonucleotide is flanked by 25 nucleotides on either side, allowing room for the UvrA dimer to bind with a footprint size of 33 bp.^29,30^ The undamaged_49 bp construct is formed from the digoxigenin-labelled oligonucleotide, and the reverse digoxigenin-labelled oligonucleotide, providing a 49 bp region of dsDNA for binding. All oligonucleotides were purchased from Eurofins Genomics, Germany.

**Table:** 

Construct name	Constituent oligonucleotides
Damaged_98 bp	Digoxigenin-labelled oligonucleotide
	Bridge oligonucleotide
	F26,50 oligonucleotide
Undamaged_98 bp	Digoxigenin-labelled oligonucleotide
	Bridge oligonucleotide
	Undamaged F26,50 oligonucleotide
Undamaged_49 bp	Digoxigenin-labelled oligonucleotide
	Reverse digoxigenin-labelled oligonucleotide

**Table:** 

Oligonucleotide name	Sequence
Digoxigenin-labelled	5′-[DIG]GCAGCGCAGGAATTCATCTGGGTGCGAGTAGGATGGGTAGTCCGACTCA-3′
F26,50	5′-GACTACGTACTGTTACGGCTCCATC[FlcdT]CTACCGCAATCAGGCCAGATCTGC-3′
Undamaged F26,50	5′-GACTACGTACTGTTACGGCTCCATCCTACCGCAATCAGGCCAGATCTGC-3′
Bridge	5′-GCAGATCTGGCCTGATTGCGGTAGCGATGGAGCCGTAACAGTACGTAGTCTGAGTCGGACTACCCATCCTACTCGCACCCAGATGAATTCCTGC-3′
Reverse digoxigenin-labelled	5′-TGAGTCGGACTACCCATCCTACTCGCACCCAGATGAATTCCTGCGCTGC-3′

### Oligonucleotide annealing

Equimolar concentrations of the constituent oligonucleotides for each construct were diluted to 1 μM in 1× TE buffer (10 mM Tris, 1 mM EDTA, pH 8). The oligonucleotide mix was heated at 95 °C for 10 minutes, before being allowed to cool slowly to room temperature.

### Protein labelling

All proteins were labelled at a 1 : 1 : 3 protein, to antibody, to Qdot ratio to ensure all proteins were only singly labelled.^31^ UvrA (expressed and purified as previously described^14^), Penta-His mouse antibody (Qiagen), and 605 nm quantum dots functionalized with goat-anti-mouse IgG (Invitrogen) are mixed to final concentrations of 200 nM, 200 nM, and 600 nM respectively and incubated at 4 °C for 1 h. Prior to loading into the flow chamber, labelled protein solutions were diluted to a final concentration of 5 nM. We have extensively shown previously that fluorescent labelling of UvrA does not affect its function.^6,14^

### Experimental setup

Prior to flow cell construction all slides and coverslips were rinsed sequentially with ethanol and water, dried under a constant stream of nitrogen, and processed for 2 min in a Harrick Plasma PDC-32G plasma cleaner before subsequent treatment with 2% (3-aminopropyl)trimethoxysilane. Flow cells are constructed as described previously.^6^ In brief, a microfluidic chamber is created from a standard glass microscope slide (with two 1 mm holes drilled 12 mm apart) and glass coverslip joined by an adhesive gasket. Polypropylene tubing is used to create an inlet and outlet tube, allowing for addition of experimental reagents to the flow chamber. This was followed by overnight incubation with 25 mg mL^−1^ mPEG_5000_ in 250 mM NaHCO_3_, pH 8.3. These flow cells were then washed with 400 μL 18 MΩ water prior to incubation with 1× ABT buffer (50 mM Tris-HCl, pH 7.5, 50 mM KCl, 10 mM MgCl_2_, 1 mg mL^−1^ BSA, 0.1% Tween-20, 0.02% NaN_3_) for 1 hour. These were then washed with 400 μL 1× ABC buffer (50 M Tris-HCl, pH 7.5, 50 mM KCl, 10 mM MgCl_2_, 0.02% NaN_3_) prior to incubation with 10 μg mL^−1^ anti-digoxigenin antibody (Roche) in 1× ABC buffer for 1 hour. Following a wash with 400 μL 1× ABC buffer, 1 nM annealed oligonucleotide in 1× ABC buffer was added and incubated for 30 min, followed by a final wash with 400 μL 1× ABC buffer. 50 μL of 5 nM UvrA-Qdot and 20 nM unlabelled UvrA in 1× ABC buffer containing 1 mM nucleotide (ATP, ATPγS or ADP) was added to the flow cell and was immediately imaged on a custom-built iSCAT microscope.

### Ratiometric and image processing

**Fig. 1:**
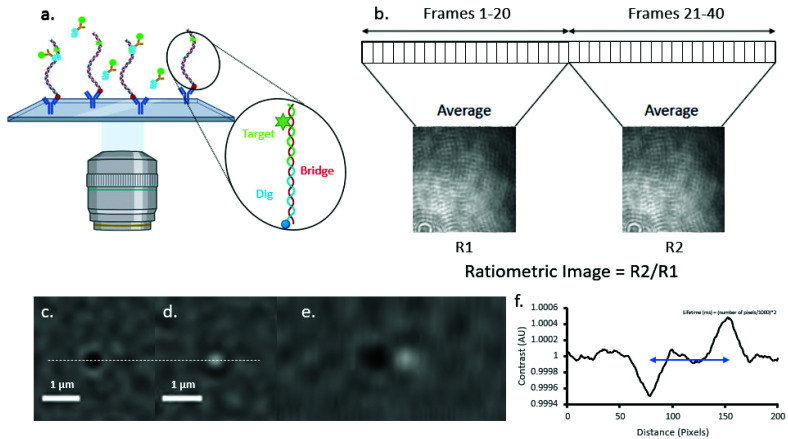
Ratiometric imaging reveals rapid binding and release events. (a) The experimental setup used in this experiment, in which surface immobilised DNA oligonucleotides are targets for UvrA-Qdot protein complexes (see ESI Fig. 3†). Binding and release events are revealed through the use of (b) ratiometric imaging, a process in which dynamic features are revealed by dividing sequential batches of *n* frames – isolating features which change between frames *n* and *n* + 1. (c) Binding and (d) release provide differential signals allowing for clear differentiation of lifetimes, as seen in (e) a kymograph of UvrA labelled with a 605 nm Qdot binding and releasing from DNA tagged with a fluorescein moiety. (f) The difference between peak contrast of binding and release provides the frames in which molecules bind and release, respectively. Lifetime is calculated from the 2 × pixel difference/1000. See also ESI Fig. 4–7.†

Ratiometric images were produced using a sliding window method, in which two sequential batches (R_1_ and R_2_) of *N* frames are averaged, normalised to their own maximum pixel intensity, and then divided (R_2_/R_1_) to produce a ratiometric image (Fig. 1b). As a result, these ratiometric images contain only features which have changed between R_1_ and R_2_. This process is repeated, advancing frame-by-frame through the whole image stack to produce a ratiometric movie. A bin size of 20 (*N*) was used for all image processing, giving a temporal resolution of 40 ms. The resulting images were convoluted with an experimentally extracted point-spread function (PSF),^25^ and a Gaussian filter (*n* = 2) was applied (see ESI Fig. 2†). All image processing was carried out using custom-written MATLAB software.

### Image and data analysis

All videos were converted into global kymographs (Fig. 1e), and a threshold applied based off the minimum measured contrast for the UvrA-Qdot complex, isolating peaks which correspond to binding and release events (single binding or release events were excluded as an accurate lifetime could not be estimated). Local minima and maxima along individual columns of the kymographs are located, and subsequently fitted to 1-D Gaussian distributions in the *X* and *Y* axis. To ensure the signal arises from a binder and not noise, the standard deviation of both axes of each peak were used to create a ratio describing the uniformity of the PSF. Any signals with a ratio of <0.9 were excluded. Lifetimes were calculated from the difference between the peak minimum and peak maximum frames (Fig. 1f). Lifetimes for each condition were collated into a cumulative decay function, and were fitted as natural logarithms to a double exponential similarly transformed into linear space. Further details are available in ESI including a more detailed description of noise analysis can be found in the ESI.†

## Results

### Interferometric scattering microscopy reveals protein–DNA interactions on a millisecond timescale

We were able to directly image the binding and release of Qdot labelled UvrA dimers from both damaged and undamaged DNA using iSCAT. This approach combines light scattering from labelled proteins in the sample with reflected light at the glass-water interface of the flow cell, leading to interference. Our *in vitro* surface-based assay (Fig. 1a) provides a target for UvrA dimer binding; digoxigenin-labelled dsDNA oligonucleotides (either labelled with a fluorescein moiety, or unlabelled) are tethered to the surface *via* an anti-digoxigenin antibody. Ratiometric imaging (Fig. 1b) allows us to clearly resolve differential signals for binding (Fig. 1c) and release (Fig. 1d) of labelled-protein from DNA by isolating dynamic features within the sample, allowing for direct visualisation of UvrA dimer–DNA interactions and lifetimes (Fig. 1e) with an achievable temporal resolution down to 1 ms. In this study, the temporal resolution is limited to 40 ms with a 20-frame bin to maximise the achievable signal-to-noise ratio when utilising Qdots as a scattering label. Since UvrA binds DNA as a dimer we henceforth use the term UvrA to describe the dimer.

UvrA uses a 3D mechanism, where it attaches to DNA and then leaves without sliding, to search DNA for damage,^6,8,14^ however, given the lifetimes of interaction and footprint size this search mechanism would not be sufficient to interrogate the entirety of an *E. coli* genome within a single division time.^6^ Additionally, it has been suggested that rather than the more efficient UvrA_2_B_2_ complex being the primary damage-sensing species within NER, UvrA instead recruits UvrB from solution upon location of damage.^8^ Therefore, to search effectively UvrA may locate damage first using an alternative, faster, mechanism of search.

**Fig. 2:**
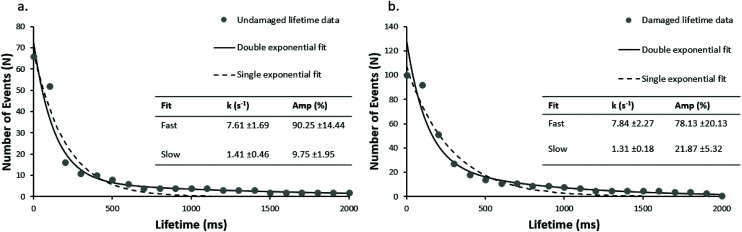
Rapid single molecule kinetics of UvrA on damage and undamaged DNA. Attached lifetimes obtained from ratiometric images were compiled into cumulative decay plots. In the absence of damage (undamaged_98 bp) (a) two clear populations are seen with a predominant fast rate constant of 7.6 s^−1^, and a slower rate constant of 1.4 s^−1^ (*r*^2^ = 0.97 *vs.* 0.71, for double and single exponential fits respectively). With fluorescein-damage (damaged_98 bp) (b), two populations (*r*^2^ = 0.96 *vs*. 0.67, for double and single exponential fits respectively) are again seen with the faster population predominant again, at 7.8 s^−1^, matching well with the fast population observed on undamaged DNA.

To investigate this, we analysed high-speed UvrA interactions with both damaged (damaged_98 bp, using fluorescein-dT, which is a well-established damage analogue^2^) and undamaged DNA (undamaged_98 bp). 5 nM Qdot-labelled UvrA was mixed with 20 nM unlabelled UvrA (resulting in a final concentration of 25 nM) in the presence of 1 mM ATP, leading to the formation of UvrA dimers that are mostly labelled with a single Qdot. Post ratiometric processing, UvrA–DNA lifetimes appear as a black spot (with a contrast similar to that of a single Qdot, consistent with the single-labelling of UvrA dimers described above), followed by a white spot in the same spatial location. Lifetimes are calculated by taking the distance between the frame in which peak contrast is reached upon binding, and the frame in which peak contrast is reached upon release. Individual lifetimes were plotted as cumulative decay functions, and fit to double exponentials following natural logarithmic transformation to linear space (Fig. 2). Single exponential fits did not adequately describe the data (Fig. 2 dotted lines). These fits provide DNA dissociation rate constants (*k*), and amplitudes corresponding to relative populations for each rate constant.

The rate constants for UvrA release on undamaged DNA (Fig. 2a) was 7.6 s^−1^ and 1.4 s^−1^, *versus* 7.8 s^−1^ and 1.3 s^−1^ on damage-containing DNA (Fig. 2b). The relative proportions of fast to slow lifetimes were 90 : 10 for undamaged DNA, and 78 : 22 for damage-containing DNA, indicating the dominant form of UvrA interactions with DNA are the fast lifetimes. It should be noted that in the absence of UvrA, Qdots alone did not attach to DNA, and in the absence of DNA no interactions were recorded between UvrA-Qdots and the passivated glass surface.

**Fig. 3:**
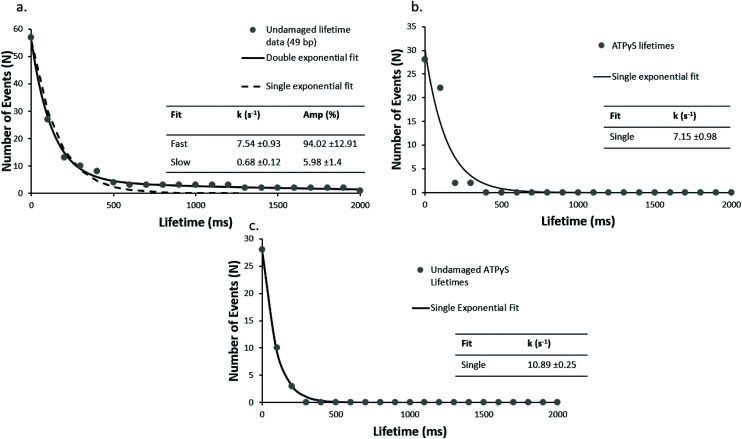
DNA length has no impact on fast lifetimes, but in the presence of ATPγS UvrA loses the slow phase. Cumulative decay plots with a shorter (undamaged_49 bp) undamaged DNA construct (a), are unchanged kinetically from the 98 bp construct suggesting the lifetimes are not limited by sliding off the DNA (*r*^2^ = 0.98 *vs*. 0.55, for double and single exponential fit respectively). UvrA interactions were also studied on (b) 98 bp damaged DNA (damaged_98 bp) and (c) 98 bp undamaged DNA (undamaged_98 bp) in the presence of 1 mM ATPγS where no slow events were seen. The observed interactions fit well to a single exponential with a detachment rate constant of 7.15 s^−1^ and 10.89 s^−1^ for damaged and undamaged DNA respectively (*r*^2^ = 0.99 and 0.91 for single exponential fit on undamaged and damaged DNA respectively).

We hypothesised that these faster events could include a localised diffusive search. Unfortunately, the architecture of this assay means a signal for movement along the DNA is not present; therefore, we reduced the length of the undamaged DNA oligonucleotide from 98 bp to 49 bp (undamaged_49 bp). As seen in Fig. 3a, the double exponential fit of the lifetime cumulative decay function for the shorter DNA construct results in a fast dissociation rate constant of 7.5 s^−1^ in good agreement with the rate constant for the 98 bp undamaged DNA (7.6 s^−1^), and a slow rate dissociation constant of 0.68 s^−1^. This suggests that the lifetime of UvrA's interaction with DNA is not limited by the DNA length. The observed slow dissociation rate constants are consistent with UvrA : DNA interactions in which UvrA hydrolyses 2 ATP molecules, with an ATP turnover rate constant of ∼1–2 ATP per s (ref. 13 and 14) – however, these are more challenging to assign due to the low number of observed slow events and limited acquisition times.

### ATP analogues dramatically reduce UvrA damage detection

**Fig. 4:**
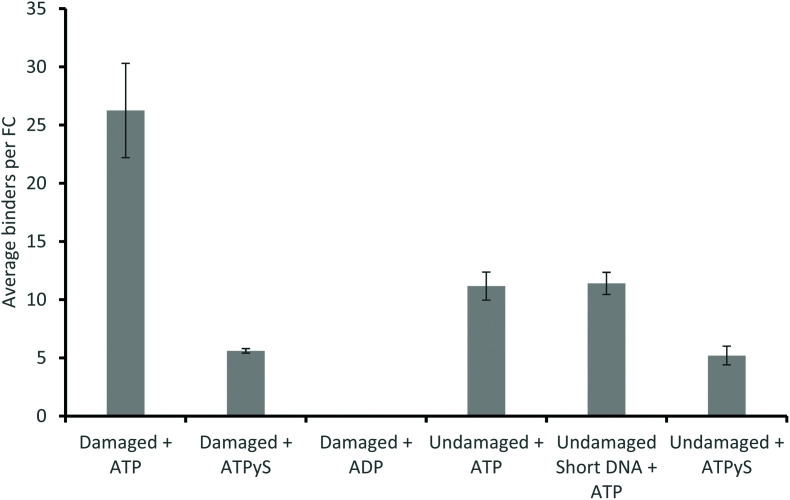
ATP hydrolysis precedes DNA binding of UvrA. A comparison of average number of binders, *n*, per flow cell in different experimental conditions. All averages were calculated from equivalent numbers of observations (10 videos per flow cell, across 6 flow cells, equal to a total 60 minutes of imaging per experimental condition). ATPγS significantly reduces the number of binders in the presence of fluorescein-damaged DNA (27 per flow cell *vs*. 6 per flow cell), and in the presence of undamaged DNA (12 per flow cell *vs*. 5 per flow cell), whilst ADP completely eradicates observable attachments to fluorescein-damaged DNA.

Use of alternative nucleotides, ATPγS (a non-hydrolysable ATP analogue) and ADP, dramatically affected the interaction of UvrA with DNA containing a fluorescein lesion (damaged_98 bp) and undamaged DNA (undamaged_98 bp). In the presence of ATPγS only fast events were seen, with a detachment rate constant of 7.15 s^−1^ (139 ms interaction lifetime) and 10.89 s^−1^ (92 ms interaction lifetime), on damaged (Fig. 3b) and undamaged DNA (Fig. 3c) respectively. Furthermore a ∼5-fold reduction in the average number of observed events in the presence of ATPγS relative to ATP with a damage-containing DNA construct was observed. This indicates that ATP turnover is necessary for loading UvrA onto the damage. In addition, the presence of ADP eradicates all interactions of UvrA with damaged DNA (Fig. 4). It has been previously found that ADP inhibits the interactions of UvrA with DNA due to its affinity for the distal UvrA ATPases,^13^ suggesting that in the presence of ADP fewer UvrA dimers are present in solution.^32^ Additionally, in the absence of ATP, no interactions were seen between UvrA and DNA.

## Discussion

Due to the vast quantity of non-target DNA, the search for damaging lesions by DNA repair systems presents a considerable challenge. A number of mechanisms have been proposed to accelerate the search,^16^ however the search is limited by the lifetime of each visit to the DNA and number of proteins. UvrA is a dimeric molecule that has been implicated in searching DNA for damage^7,33^ in bacteria. However, its long attached lifetime,^6,8,14^ small footprint,^30^ and relatively low abundance^34^ suggest it cannot search the entire genome before cell division. Here we use interferometric scattering microscopy (iSCAT) to determine the lifetime of UvrA's (the term UvrA is used here to describe the dimer form) interaction with DNA. We find UvrA's interactions consists of two phases, one consistent with that expected from ATPase measurements,^13,14^ and a faster phase with an average interaction time of 130 ms. This is supported by our observation that UvrA's lifetime in the presence of the non-hydrolysable analogue ATPγS eliminates the slower phase, but not the rapid lifetimes. Furthermore, the length of the target DNA molecule does not affect this rapid lifetime suggesting UvrA exclusively uses a 3D search rather than a limited 1D search. Altogether, these data suggest a potential new mechanism through which UvrA searches the genome efficiently using an ATP-independent rapid search, followed by ATP turnover at suspected damage sites.

### UvrA alone is able to effectively search the *E. coli* genome *via* a 3D search mechanism

**Fig. 5:**
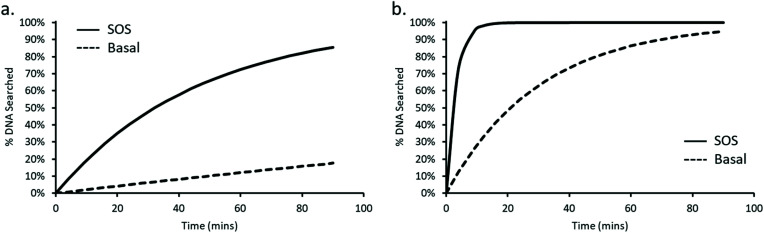
Predicting the percentage of *E. coli* genome searched by UvrA in a single division cycle. The percentage of the *E. coli* genome searched by UvrA is plotted against time, for an average lifetime of 2 s, (a) showing that after 90 minutes only 18% of the genome is searched by basal levels of UvrA (dashed line) *vs*. 86% at SOS levels (continuous line). Predicting the genome search time for the faster, 130 ms, lifetime measured here (b) suggests 95% of the genome is searched within 90 minutes without SOS levels of UvrA (dashed line). Whereas 95% of the genome is searched after only ∼9 minutes at SOS protein levels (continuous line).

UvrA interacts statically with DNA leading to binding events with a lifetime of ∼1–3 seconds.^8,14^ It has been previously suggested that the formation of the UvrA_2_B_2_ complex on DNA is driven by recruitment of UvrB from solution by UvrA bound to sites of damage.^8^ This would suggest that the time taken for locating damage by UvrA is a key factor within this process. To calculate how this lifetime relates to the proportion of the *E. coli* genome that can be searched by UvrA within a single *E. coli* division cycle we employed a simple exponential distribution of the Poisson process:% of genome searched = 1 − *e*^(−*τx*)^where *x* is time, and *τ* is the proportion of DNA searched by the total number of UvrA dimers within a cell per second:
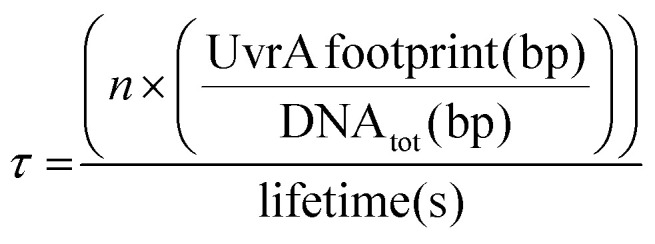
where *n* is the total number of UvrA dimers present within the cell, the UvrA footprint is 33 bp,^30^ lifetime is the duration of interaction, and DNA_tot_ is the total amount of DNA that needs to be searched. The basal level of UvrA within a cell has been estimated from 9–129 depending on conditions,^8,35–37^ here we take the canonical measurement of 20 ^35^, this is equivalent to 10 dimers. During the SOS response UvrA is upregulated, resulting in ∼200 copies of UvrA equivalent to 100 dimers. With the average division time of an *E. coli* cell at 25 °C being 90 minutes,^38^ we can see in Fig. 5a that UvrA is unable to search the majority of DNA within an *E. coli* cell, reaching a total coverage of ∼18% going up to ∼86% during SOS over the 90-minute period. This highlights the vast inadequacy of this 3D search mechanism with a lifetime of 2 seconds.

However, incorporation of the rapid 130 ms lifetime that we have observed leads to a dramatic increase in the proportion of DNA searched by UvrA within a single division cycle, allowing UvrA to reach 95% coverage of the *E. coli* genome within ∼90 minutes (Fig. 5b). Upon induction of the SOS response this search time drops again, resulting in 95% coverage of the *E. coli* genome being reached in ∼9 minutes. We previously thought that the 3D search mechanism of UvrA would prove wholly incapable of providing an efficient search mechanism within *E. coli*^6,39^ – however these results indicate a potentially larger role played by UvrA alone during the early stages of DNA damage detection within NER.

This process could work in combination with the ability of UvrB to form the UvrA_2_B_2_ dimer,^6,40^ collapsing the 3D search of UvrA alone into a 1D search by the UvrA_2_B_2_ complex. This 1D search is the most efficient form of damage detection within NER^6^ – however, it has been noted that during *in vivo* experiments the vast majority of UvrB remains diffusive within solution (∼90%).^8^ It is possible that the rapid search by UvrA bridges these two observations and allows for rapid search of the DNA by UvrA, leading to the recruitment of UvrB from solution upon location of DNA damage. However, further work needs to be carried out to understand how these processes are connected. Together these search mechanisms could provide an effective combination of 3D and 1D searches allowing for an entire *E. coli* genome to be effectively searched by a small number of proteins within a single division cycle.

### UvrA adopts a low energy cost search mechanism

As detailed above, it has been previously found the UvrA interactions with DNA lead to static binding events with a lifetime of ∼2 seconds along with an ability to hop to nearby DNA molecules.^6,8,14^ During this period, 2 molecules of ATP would be consumed,^13,14^ resulting in a high energetic cost per interaction.

We confirmed that the rapid interactions we have observed do not consume ATP through the use of the non-hydrolysable ATP-analogue ATPγS, which eliminates slower lifetimes. The consequence of a non-ATP consuming process as a first means to detect DNA abnormalities results in a dramatically reduced energetic cost for the cell. Longer, ATP-consuming, interactions were detected by iSCAT in both the presence and absence of damage, consistent with previous studies,^8,13,14^ however their prevalence was much lower. This suggests that the first step in damage detection is performed by UvrA without requiring ATP. We propose that this cursory check is followed by an ATP-requiring second check, before the next ATP-consuming event that loads UvrB.^5,13,14,41^ This means, based on the UvrA ATPase turnover rate of 1 ATP per UvrA-monomer per second, that within a typical *E. coli* doubling time at 25 °C of 90 minutes,^38^ the entirety of the genome, 4.6 million base pairs, could be searched with a maximum energetic cost of ∼110 000 ATP molecules; however since only 10% of UvrA molecules enter the slow phase the better estimate of energetic cost is ∼11 000 ATP molecules equivalent to 3.3 × 10^5^ kJ mol^−1^.

### The role of ATP in the fast-association rate of UvrA

The structure of DNA is altered by the presence of a lesion, with more distorting lesions being excised with greater efficiency.^9^ UvrA plays a clear role in this recognition process since its affinity for DNA has been shown to be greater in the presence of damage.^29,33^ Here, we show that UvrA binds to DNA ∼2-fold more frequently in the presence of damage, suggesting that the increased affinity of UvrA for damage is mediated by the attachment rate constant. This would suggest that UvrA binds to DNA in a conformation that stabilizes the damaged DNA (conformational selection) rather than through induced fit. The correct nucleotide occupancy of the ATP-binding sites on UvrA appears crucial to permitting damage detection and even DNA binding. UvrA with ATPγS was found to bind to damaged and undamaged DNA with much lower frequency than UvrA with ATP. Indeed, the level of binding to DNA was lower than expected if only the slow phase events were absent (which was observed). This suggests ATPγS drives UvrA in a lower affinity form for DNA binding, but not as low as in the absence of ATP which showed no binding in these experiments. Recent studies of UvrA's ATPase have indicated that the proximal and distal ATP-binding sites attain an asymmetric nucleotide bound state (proximal-ATP : distal-ADP) prior to interacting with DNA; upon meeting damage the distal site is activated.^13^ In an elegant crosslinking study, ATP hydrolysis at this distal site was linked to the movement of the third zinc finger (zinc finger 3) over the DNA,^42^ that was previously implicated in damage recognition.^33,43^ Therefore, with ATPγS UvrA cannot assume the conformation needed to select for damaged DNA. Furthermore, once damage is located, ATP hydrolysis at the distal site would be needed to confirm its presence before recruiting UvrB.

### A model for DNA damage detection by UvrA

**Fig. 6:**
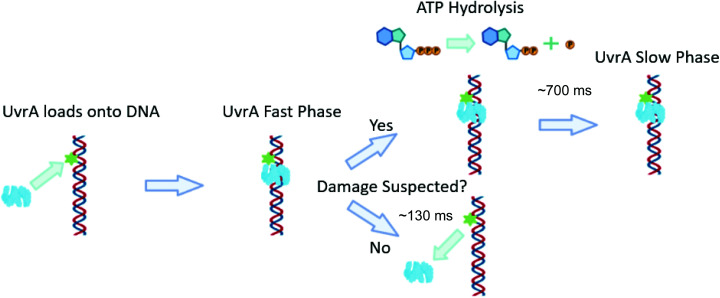
A model for early NER damage detection by UvrA. UvrA randomly binds to DNA with a footprint of 33 base pairs. If DNA damage is not found at this site UvrA dissociates with a lifetime of ∼130 ms, and no ATP is consumed. If DNA damage is suspected†, the UvrA dimer hydrolyses ATP (∼700 ms) to confirm the presence of damage^14^ followed by loading of UvrB and subsequent DNA repair. This figure is created without UvrB, in its presence UvrA may be bound to UvrB throughout the search phase. Created with BioRender.com.

Here we present a model for the early, ATP-independent, damage detection of UvrA (Fig. 6). UvrA utilises a 3D search mechanism and upon binding DNA remains statically bound for ∼130 ms. During this time, no ATP is hydrolysed but the DNA is inspected, possibly by the movement of zinc finger 3.^42^ If no damage is detected the UvrA dimer is able to dissociate from the DNA, from here it returns into solution with an increased probability of reassociating on another nearby DNA strand due to the high-local concentration of DNA within a cell. However, if zinc finger 3 detects a site of suspected damage, UvrA hydrolyses ATP to lock it onto DNA for subsequent damage verification, which if detected leads to UvrB loading, in preparation for subsequent processing by downstream NER proteins. This offers a much more parsimonious model of DNA damage recognition by the NER apparatus.

## Conclusions

Using high speed imaging we have discovered a previously unseen component of UvrA's search for damage. UvrA rapidly binds for ∼130 ms without consuming ATP before releasing from DNA. These interactions probe for damage and offer a first step in a kinetic proofreading mechanism of damage detection. By employing an initial rapid search, this enables native levels of UvrA to reliably scan the entirety of an *E. coli* genome during a single division cycle without triggering the SOS response. We propose a model for early damage detection for NER by UvrA and detail the close association of ATP hydrolysis for further damage verification and subsequent UvrB recruitment.

## Conflicts of interest

There are no conflicts to declare.

## Supplementary Material

NR-014-D1NR06913F-s001
