# Supplementary material for: Single molecule iSCAT imaging reveals a fast, energy efficient search mode for the DNA repair protein UvrA
Source: Nanoscale. 2022 Mar 21;14(13):5174–84. doi: 10.1039/d1nr06913f (PMC8969456; doi:10.1039/d1nr06913f)
Supplement: NR-014-D1NR06913F-s001 [file NR-014-D1NR06913F-s001.pdf]

Electronic Supplementary Material (ESI) for Nanoscale.
This journal is © The Royal Society of Chemistry 2022

Supplementary materials: Single molecule iSCAT imaging reveals a fast, energy efficient search mode for the DNA repair protein UvrA

Robert J Charman[1] and Neil M. Kad[1,2]

[1]School of Biological Sciences, University of Kent, Canterbury CT2 7NH, UK

[2]To whom correspondence should be addressed. Tel: +44 (0)1227 816151; n.kad@kent.ac.uk

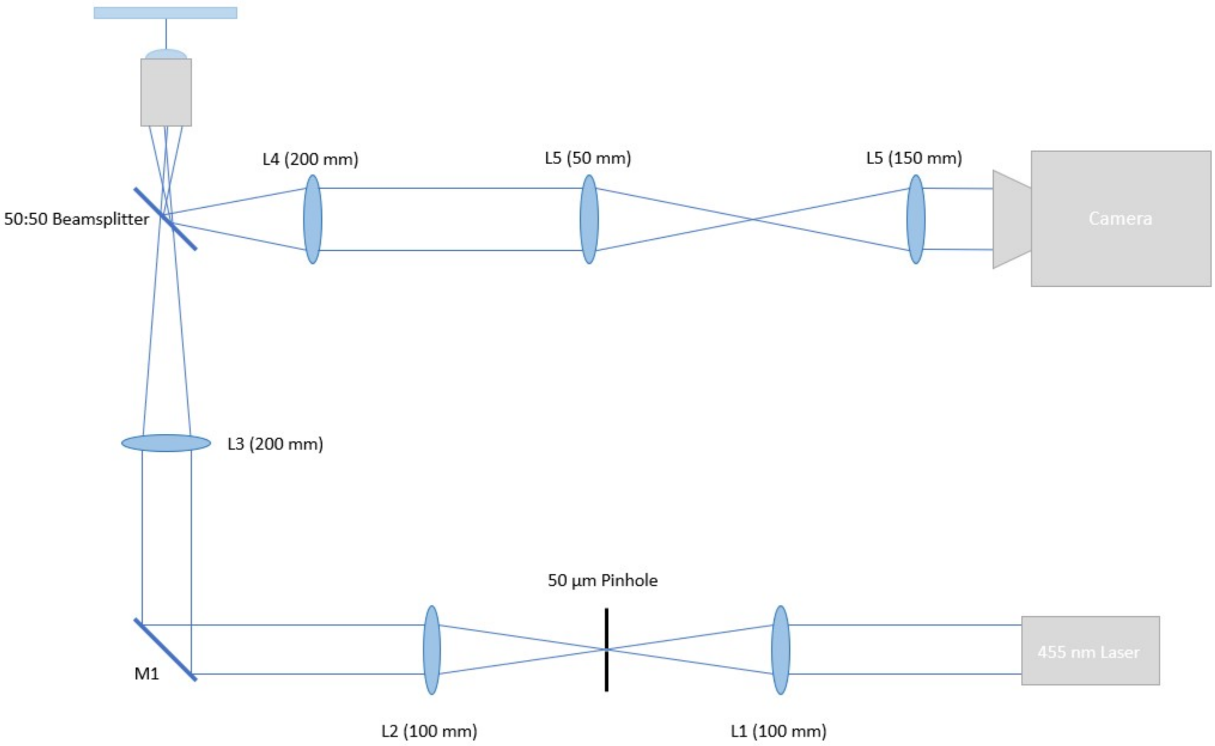

Supplementary Fig 1. – Schematic of iSCAT microscope

The output of a 300 mW 445 nm diode laser (Roithner-Lasertechnik ULV-445-300), modulated at 20 kHz, is focused through a 50 μm pinhole filter (ThorLabs P50HD) via a 100 mm plano-convex NBK-7 lens (L1). The beam is recollimated via a 200 mm plano-convex NBK-7 lens (L2), before being focused into the back focal plane of the objective (Nikon CFI Apochromat TIRF 60x Oil Immersion Objective Lens, NA 1.46) by a 200 mm plano-convex NBK-7 lens (L3). The beam is redirected to the objective using a 50:50 beam splitter, providing separation between the illumination and detection pathways. The resulting reflected and scattered fields are collected by the objective, before travelling back through the 50:50 beam splitter, and undergo collimation by a 200 mm plano-convex NBK-7 lens (L4). The reference and scattered waves pass through a telescope constructed from a 50 mm plano-convex NBK-7 lens (L5) and a 150 mm plano-convex NBK-7 lens (L6), resulting in a final magnification of 180x, and is focused onto the sensor of a Flir Grasshopper USB3 CMOS camera (Point Grey Research, GS3-U3-23S6M-C).

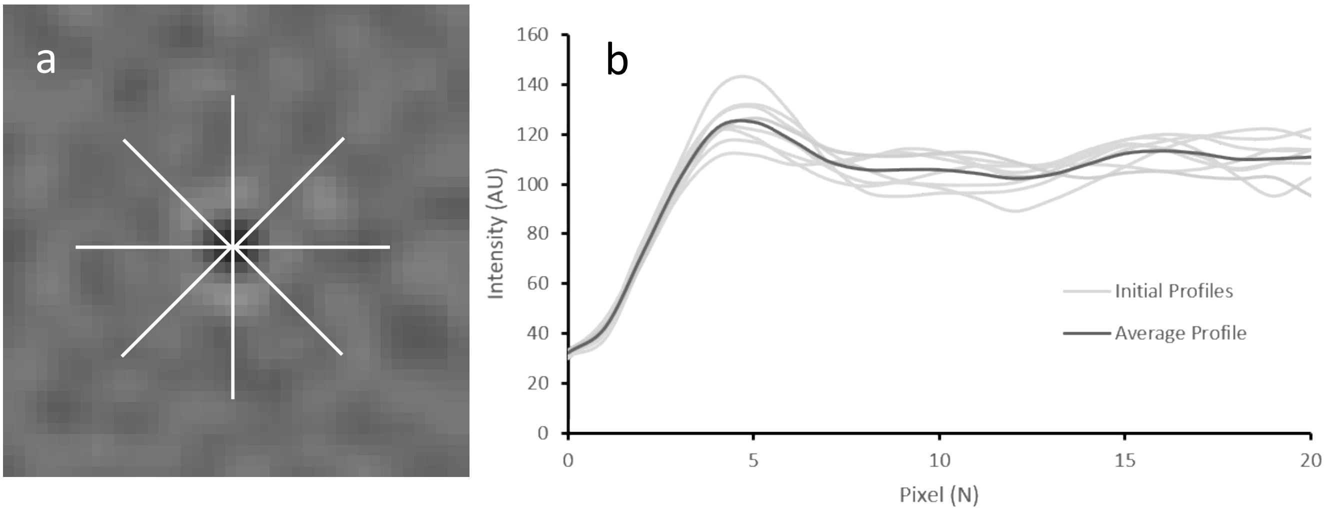

Supplementary Fig 2. – iSCAT PSF Estimation

iSCAT PSFs are estimated as previously described (Taylor et al., 2019). (a) A radial series of 360 intensity profiles with a length of 20 pixels are taken, with one degree of separation between each, extending out from the centre of the binder PSF. (b) The profiles often display differing intensities due to inhomogeneities in the local area, therefore these profiles are average to produce an accurate estimation of the true radial profile of the iSCAT PSF. This is then reprojected as a 2D PSF and used for experimental image convolution.

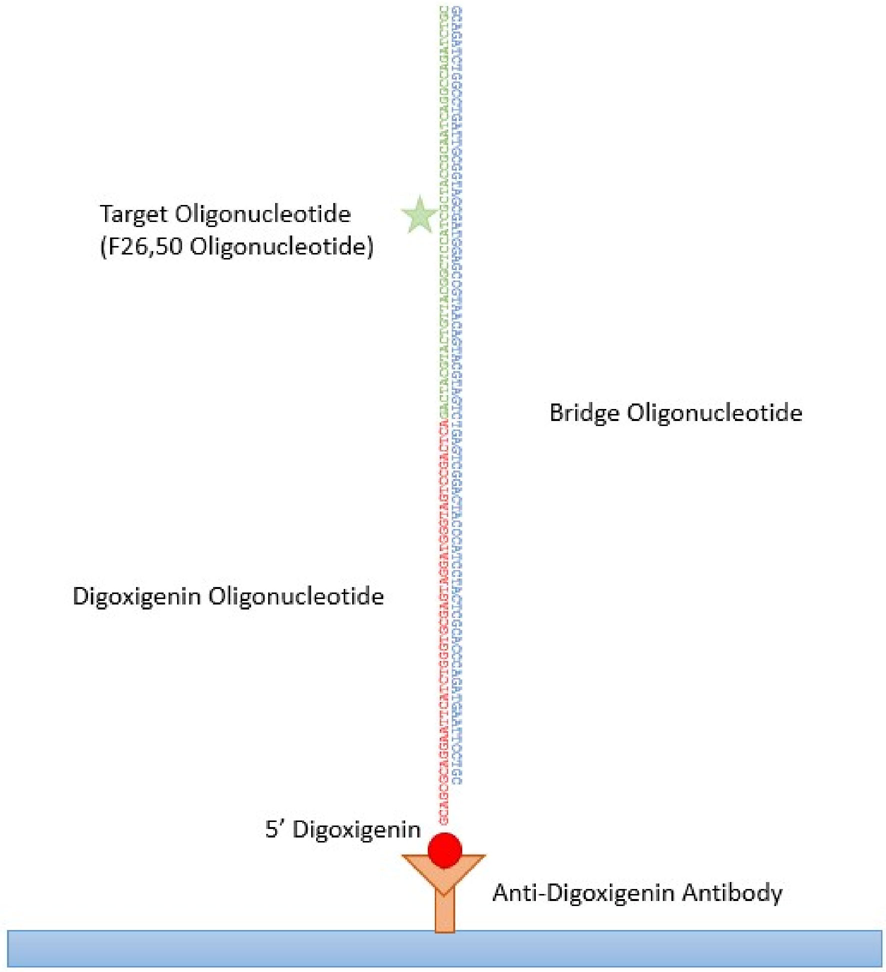

Supplementary Fig 3. – DNA Construct Design

The rationale behind the design of the DNA construct within this assay is to provide a flexible base for studying protein-DNA interactions – providing the facile ability to introduce synthetic lesions, specific sequences, and specific structures into the experimental construct. The design of the construct involves 3 oligonucleotides: the Digoxigenin Oligonucleotide, the Bridge Oligonucleotide, and the Target Oligonucleotide. The Digoxigenin Oligonucleotide allows for surface-tethering of the construct to a coverslip. The Bridge Oligonucleotide is complementary to both the Digoxigenin Oligonucleotide, and the Target Oligonucleotide and bridges both to allow for a complete dsDNA construct. The Target Oligonucleotide can be altered to include any required target site, here we use a fluorescein-labelled oligonucleotide as a target for UvrA as it is a well-established DNA damage analogue for prokaryotic NER.

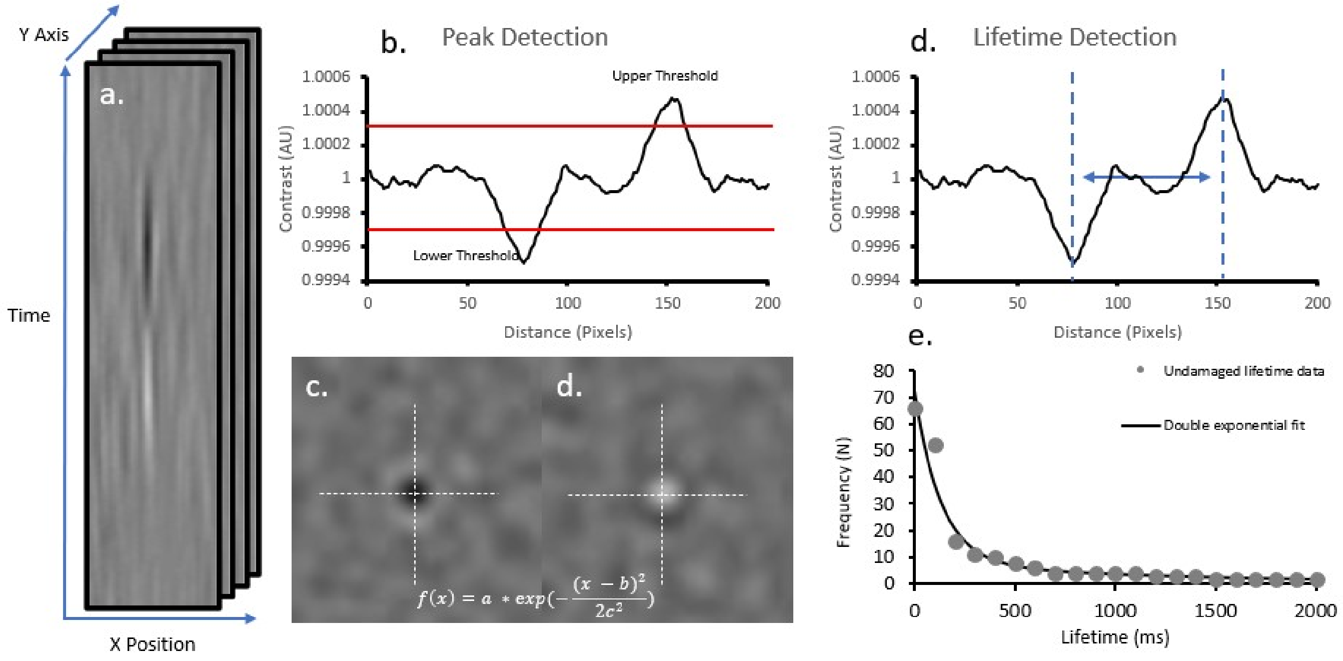

Supplementary Fig 4. – Lifetime detection and analysis

*The image stack undergoing analysis is first converted into a global kymograph (**a**) in which the x-axis represents one row of pixels from the ratiometric image, and the y-axis represents time – whilst the y-axis of the ratiometric image stack is converted into the z-axis of the global kymograph (i.e., every frame of the global kymograph represents how one row of pixels from the ratiometric video changes over time). For each pixel along the x-axis an intensity profile is plotted (**b**), and local points of maxima and minima are identified, with a lower and upper threshold being applied for binding and release respectively. These threshold values are calculated based on experimentally extracted contrasts for individual Qdots. Upon location of points of maxima and minima, the PSFs for the particles at those times are extracted (**c**), and a 1D Gaussian is fit in the X and Y axes for both binding and release PSFs. To assess the ellipticity of the particle causing the binding/release signal, a ratio of the smallest to the largest c value (the spread of the Gaussian fit) is created, and signals with a ratio < 0.9 are rejected as arising from noise. The lifetime of confirmed binding and release events (**d**) is then calculated as the distance between the frames at which the local minima, and subsequent local maxima are reached. Lifetimes are then collated and used to create a cumulative decay function, which subsequently undergoes a natural logarithmic transformation. This is then fit to a double exponential in linear space (**e**) to extract the dissociation rate constant and respective amplitudes of the two populations.*

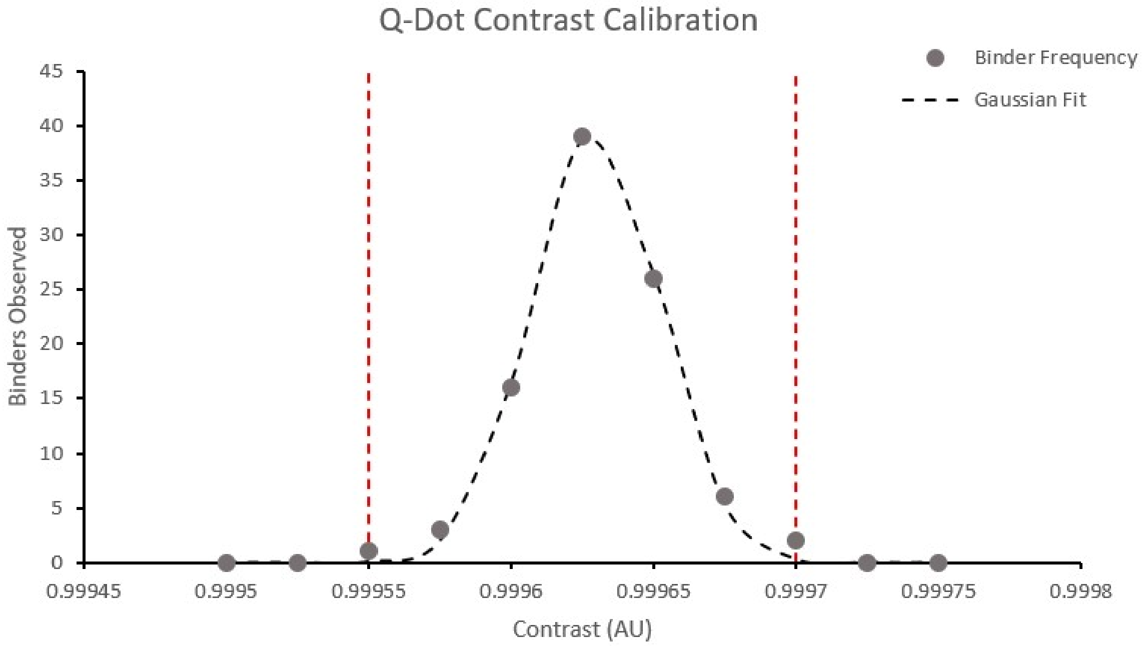

Supplementary Fig 5. – Qdot Contrast Calibration and Threshold Calculation

Histogram of observed contrasts for individual Qdots landing on the surface of a glass coverslip, from this distribution we are able to set the threshold values for lifetime detection. The upper and lower thresholds are taken as the maximum and minimum detected contrasts for single Qdots.

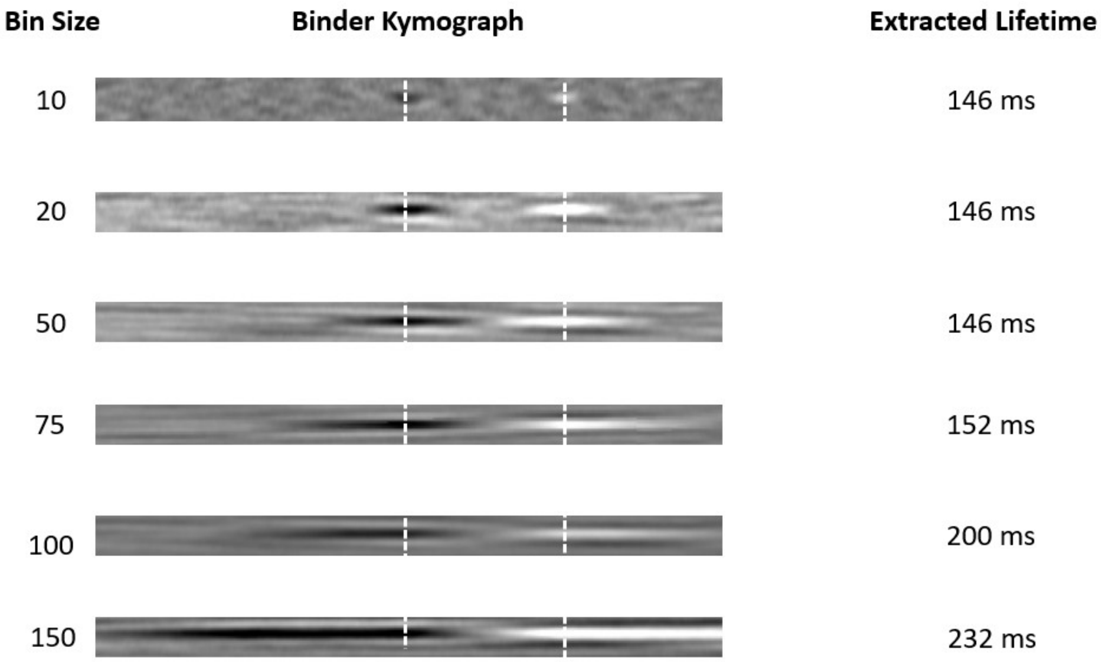

Supplementary Fig 6. – The effect of ratiometric bin size on attachment lifetimes

A binding and release event of UvrA-Qdot was processed using a range of ratiometric bin sizes (10-150), with the resulting lifetimes extracted. It can be seen that large bin sizes can result in extension of the resulting lifetime. To ensure lifetimes were not extended, we analysed a subset of videos with multiple bin sizes (5-20), to ensure that the observed lifetimes were not modified.

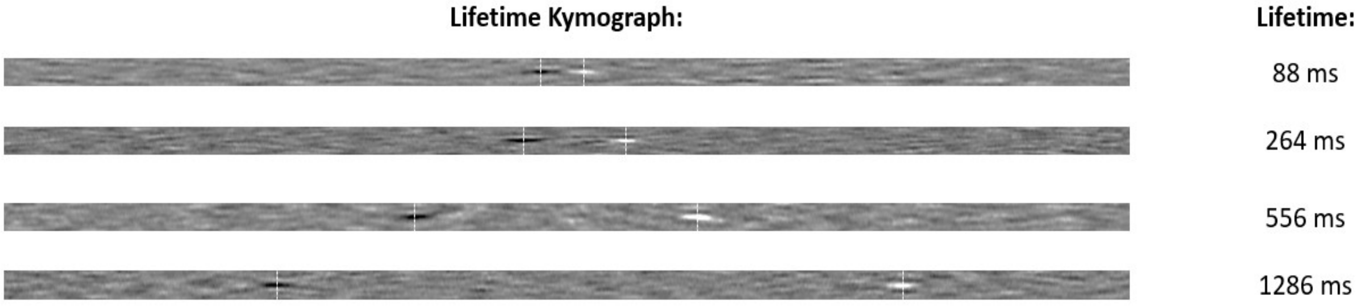

Supplementary Fig 7. – Example kymographs of binding and release events with varying lifetimes

Kymographs showing of a range of binding (black spot) and release events (white spots) with different lifetimes (88 ms – 1266 ms).

Noise Analysis and Achievable Signal-To-Noise Ratio

The achievable signal-to-noise ratio for a single Qdot was calculated using:

$$SNR = \frac{\mu_{signal} - \mu_{background}}{\sqrt{\sigma_{signal}^{2} - \sigma_{background}^{2}}}$$

Where μ_{signal} and $\mu_{background}$ are average signal from a Qdot and the background respectively (both extracted from 10x10 pixel area), and σ^2_{signal} and $\sigma^2_{background}$ are the variances of the signal and the background noise respectively (1, 2). With a 20 ratiometric bin size we achieve a signal-to-noise ratio of 11.01 for a single Qdot.

To further understand the effect of bin size on the background noise level, and subsequent achievable signal-to-noise ratio when utilising Qdots as a scattering label, a representative video was processed with a range of bin sizes and the resulting background noise level was calculated (Supplementary Fig. 8). It can be seen that the resulting σ noise follows a linear trend when plotted in logarithmic space which matches well with the shot-noise limited imaging regime of iSCAT. As bin sizes continue to be increased external sources of noise, such as mechanical instabilities and drift, begin to be incorporated within the resulting frames which leads to deviation from this linear profile. This decrease in noise results in a proportional increase in achievable signal-to-noise, at the expense of achievable temporal resolution.

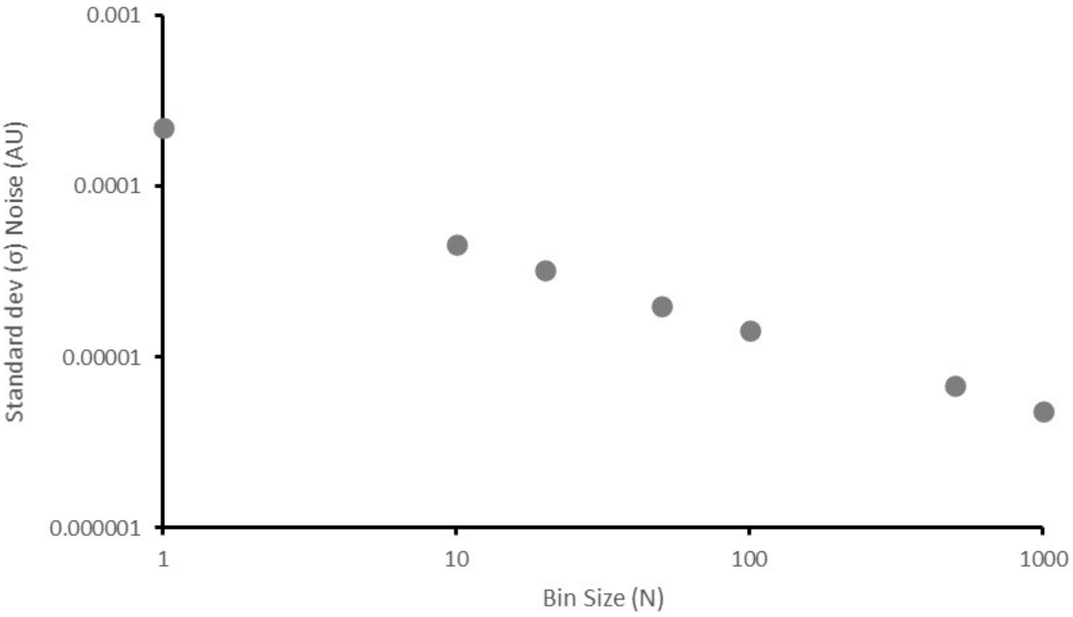

Supplementary Fig 8. – The effect of bin size on background noise

Images were processed with a range of bin sizes, and the standard deviation (σ) of the background noise was calculated. This reveals the shot-noise limited imaging regime of iSCAT microscopy, forming a linear relationship on a logarithmic scale. Continual increases in bin size theoretically leads to continual decreases in the level of background noise, however at larger bin sizes external sources of noise such as mechanical instability/drift is incorporated leading to deterioration of the images and deviation from this linear relationship.

Efficacy of Surface Passivation

To ensure that the surface-passivation applied throughout our experiments is effective in limiting non-specific surface binding and release of either Qdots alone or UvrA-Qdot complexes, control experiments (with the same experimental protocol described in the main text) were performed in the absence of DNA to assess the level of non-specific binding to the surface for both these species. In both cases whilst a small amount surface binding was observed (< 1 event per flow cell), no subsequent release events were observed within the experimental acquisition periods. Since we only include observations that both bind and release, this would exclude all of these rare non-specific interactions from data analysis.

References

1. Kubitscheck U, Kückmann O, Kues T, Peters R. Imaging and Tracking of Single GFP Molecules in Solution. Biophys J. 2000 Apr;78(4):2170–9. Available from: https://linkinghub.elsevier.com/retrieve/pii/S0006349500767646

2. Koyama-Honda I, Ritchie K, Fujiwara T, Iino R, Murakoshi H, Kasai RS, et al. Fluorescence Imaging for Monitoring the Colocalization of Two Single Molecules in Living Cells. Biophys J. 2005 Mar;88(3):2126–36. Available from: https://linkinghub.elsevier.com/retrieve/pii/S0006349505732744
